# Design and Performance of a Multi-Point Scan Confocal Microendoscope

**DOI:** 10.3390/photonics1040421

**Published:** 2014-11-20

**Authors:** Matthew D. Risi, Houssine Makhlouf, Andrew R. Rouse, Anthony A. Tanbakuchi, Arthur F. Gmitro

**Affiliations:** 1 College of Optical Sciences, University of Arizona, 1630 E. University Blvd., Tucson, AZ 85721, USA; 2 Department of Medical Imaging, College of Medicine, University of Arizona, PO Box 245067, Tucson, AZ 85724, USA

**Keywords:** confocal microscopy, microendoscopy, endomicroscopy, optical biopsy, multi-point imaging, Nipkow

## Abstract

Confocal fluorescence microendoscopy provides high-resolution cellular-level imaging via a minimally invasive procedure, but requires fast scanning to achieve real-time imaging *in vivo*. Ideal confocal imaging performance is obtained with a point scanning system, but the scan rates required for *in vivo* biomedical imaging can be difficult to achieve. By scanning a line of illumination in one direction in conjunction with a stationary confocal slit aperture, very high image acquisition speeds can be achieved, but at the cost of a reduction in image quality. Here, the design, implementation, and experimental verification of a custom multi-point aperture modification to a line-scanning multi-spectral confocal microendoscope is presented. This new design improves the axial resolution of a line-scan system while maintaining high imaging rates. In addition, compared to the line-scanning configuration, previously reported simulations predicted that the multi-point aperture geometry greatly reduces the effects of tissue scatter on image quality. Experimental results confirming this prediction are presented.

## 1. Introduction

Confocal microscopy is an established technique for imaging thick biological specimens. Confocal microscopes operate by scanning an illumination pattern across an object. The return signal is de-scanned and imaged to a conjugate plane that contains an aperture corresponding to the scanned illumination pattern. The aperture reduces the light throughput from out of focus planes in the sample, which is the basis for the optical sectioning property of confocal imaging systems. Confocal microscopes have been adapted for *in vivo* use by integrating them into portable instruments called confocal microendoscopes (or confocal endomicroscopes). Such systems are one of a class of techniques described as “optical biopsy” [1–7] that enable non-destructive *in situ* evaluation of tissue for real-time disease diagnosis.

Confocal microendoscopes typically use either a single mode fiber or an imaging fiber bundle to relay the illumination and fluorescence, or backscattered light, to and from the endoscope tip. In single fiber systems, the field-of-view is covered by either physically scanning the fiber [8] or by a miniaturized optomechanical scanner at the distal end of the probe [9–12]. When fiber bundles are used, scanning can be done at the proximal end of the fiber without the need for a miniaturized scanning mechanism.

In traditional confocal imaging systems, the illumination is a point, the confocal aperture is a pinhole, and the image is built up by raster scanning the illumination point across the sample in two dimensions. While this configuration can approach ideal imaging performance [13], it has until relatively recently been impractical for real-time *in vivo* biomedical imaging, which requires high frame rates to avoid image degradation due to object motion. Advances in resonant galvanometer technology have made point-scanning systems faster, but these scanners add complexity and cost to the system, and still remain the limiting factor for the maximum imaging frame-rate achievable. Because of the short per pixel dwell times of these high frame-rate systems, sensitive photomultiplier tubes (e.g., gallium arsenide phosphide PMTs) with high quantum efficiency are employed. Additionally, the non-linear velocity of sinusoidally-driven resonant galvanometers means that non-uniform temporal sampling is required to achieve uniform spatial sampling. This can be accomplished with additional hardware that measures the actual scan position and provides appropriately timed trigger signals to the digital sampling circuitry. The changing direction of the scan from line to line also requires specialized read/write buffers, or software compensation.

While resonant galvanometers, which must operate at a fixed resonance frequency, enable the realization of fast point-scan confocal systems, they are not suitable for multispectral imaging, where scan rates must be slowed down to allow recording and readout of dispersed light across an array detector. Another non-resonant scanning mechanism can be included for this purpose, but this adds additional components, complexity, and cost to the instrument.

Rather than increasing the speed of a point-scanning mechanism, it is possible to achieve real-time or faster frame rates in a confocal scanning system by parallelizing the illumination and detection paths. One straightforward method to accomplish this is by line-scanning. This approach uses a line of illumination, a confocal slit aperture, and builds up an image by scanning the illumination across the sample in one dimension, using any of variety of scanning techniques including a galvanometer mirror [14–16], acousto-optic scanner [17], polygon scanner [18], or spectral dispersion [19]. Line-scan systems are capable of imaging at very high frame rates [17]. However, their inherent axial resolution (optical sectioning performance) is inferior than that of point-scan systems [13]. In addition, Monte Carlo simulations have shown that the imaging performance of line-scan systems is strongly dependent on the light scattering properties of the sample [20]. As a result, line-scan imaging performance in turbid media, such as biological tissue, is significantly reduced compared to point-scan systems.

Multi-point imaging is an approach designed to overcome the inherent performance limitations of line illumination imaging while still enabling high frame rates. One well-known form of multi-point imaging is a Nipkow disc, which rapidly samples the full field of view with a series of point apertures arranged in a spiral pattern on a two-dimensional disk. However, Nipkow disc systems require switchable emission filters or multiple detection paths to accomplish multi-spectral imaging [21] and can only do so with relatively poor spectral resolution. To perform multi-point imaging and maintain a geometry compatible with multi-spectral imaging, we developed a novel approach to multi-point scanning confocal microscopy that functions as a simple modification to a slit-scanning system.

This paper describes the basic multi-point concept and its realization in a fiber bundle based confocal microendoscopic system. Imaging results comparing the multi-point and line-scan methods are shown along with measurements validating the improved performance of the multi-point approach for imaging in scattering media.

## 2. Multi-Point Imaging

A way to implement multi-point imaging is to rapidly move a linearly spaced array of illumination points along a line. This acts as the fast scan axis and creates an effective line of illumination, which is simultaneously, but more slowly, swept in the perpendicular direction, as is done in a line-scan system. A matching multi-point aperture is required for confocal imaging. We have implemented this approach using a spinning disk that consists of an array of slit apertures.

**Figure 1:**
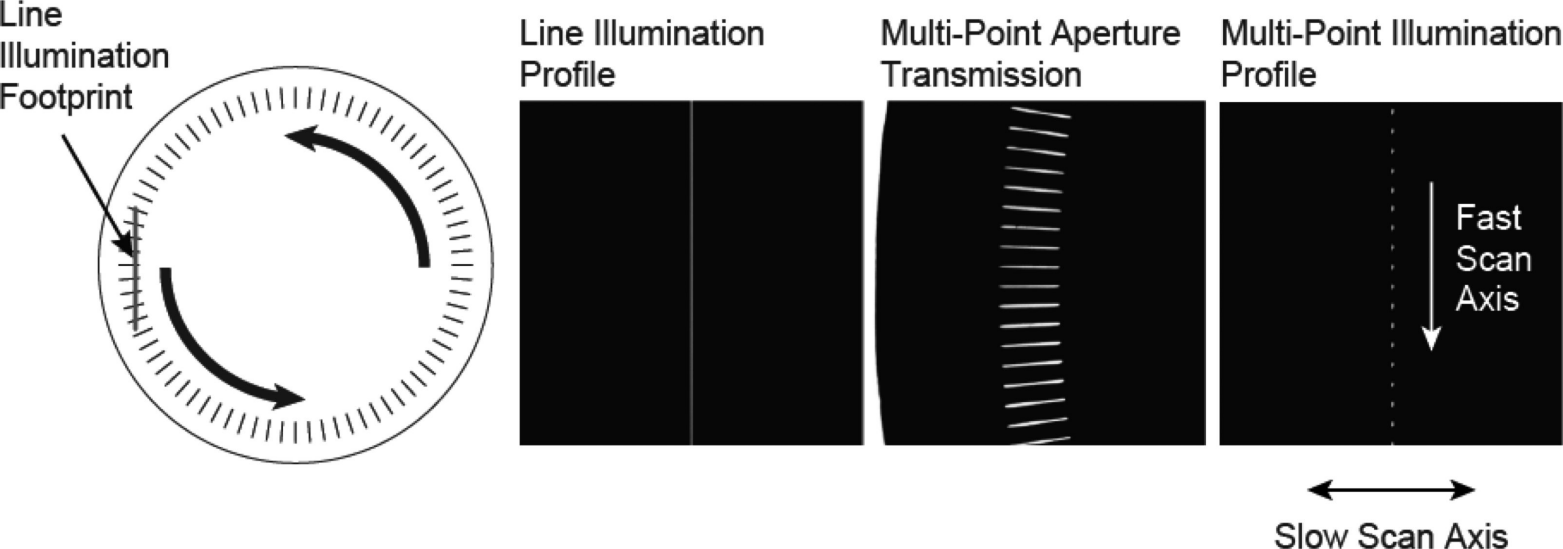
Left, a diagram of the custom rotating aperture showing the intersection of the radial slit array with line of illumination. The intersection of the line illumination profile with the transmission of the multi-point aperture creates a series of illumination points which move in the fast scan direction as the aperture is rotated. This creates an effective line of illumination, which is swept across the object in the slow scan direction using a single-axis galvanometer mirror.

The slits in the disk are oriented along the radial direction, and exist over a finite radial range (from *R_max_* to *R_min_*). They have a fixed width, and are separated by a fixed angle, Δ*ϕ*. The width of the slits, *w*, their angular separation, Δ*ϕ*, and their average radius, *R_ave_* = (*R_max_* – *R_min_*)/2, define the duty cycle of open to opaque regions as one moves azimuthally around the disk (duty cycle ≅ *w*/(*R_ave_*Δ*ϕ*)). To create a linear array of illumination points, a line of illumination is imaged onto the disk to intersect with the slit apertures as shown in Figure 1. This creates an array of illumination points that is then relayed to the object. As the disk spins, the intersection points move along the line of illumination, creating a moving multi-point illumination pattern, which serves as the fast-scan axis of the system. In detection, the combination of the rotating multi-point mask with a confocal slit aperture creates an effective multi-point confocal aperture that matches the illumination pattern.

The full system field of view is covered using a single galvanometer mirror to scan the effective line illumination in the direction perpendicular to the motion of the points. A two-dimensional image can be obtained using either a linear detector array, which reads out lines of image data as the galvanometer mirror scans across the object field, or by using a second synchronized galvanometer mirror that re-scans the light transmitted through the confocal aperture onto the image plane of a two-dimensional detector. By simply removing the rotating disk from the light path, the system reverts to a conventional line-scan confocal instrument. The multi-point approach with synchronized scan mirrors and a two-dimensional detector is also compatible with our implementation of multi-spectral imaging.

**Figure 2:**
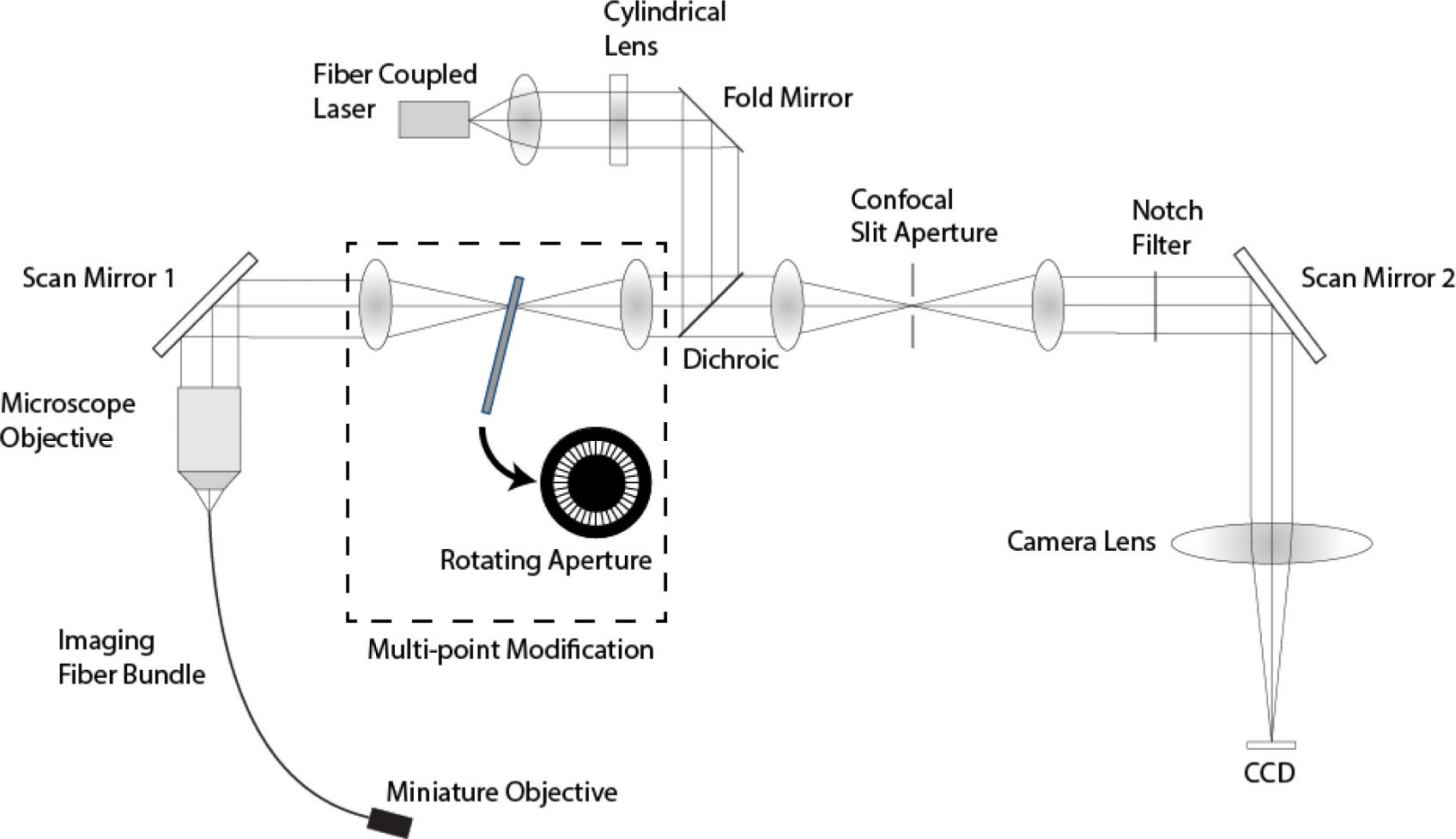
System diagram of the modified line-scan confocal microendoscope system.

To demonstrate this concept of multi-point scanning, we modified a previously reported line-scan fluorescence confocal microendoscope with multi-spectral imaging capability [1–3,22]. Figure 2 shows a diagram of the line-scan system with the multi-point modification. In this system, anamorphic illumination optics convert a 488 nm collimated laser source into a line of excitation light at the proximal end of a 30000 element imaging fiber bundle. The bundle has an active area of 720 μm and a core center-to-center spacing of 4 μm. The line of illumination is scanned across the fiber via a single-axis galvanometer mirror (Scan Mirror 1). At the distal end of the imaging fiber bundle, the line illumination is imaged into the sample via a custom miniature objective lens [23]. The return fluorescence light is de-scanned by the galvanometer mirror and imaged onto a confocal slit aperture. The light passing through the aperture is re-scanned by a second galvanometer mirror (Scan Mirror 2) and imaged onto a 2D CCD detector. Full field scanning occurs within the integration time of the CCD camera to create a 2D fluorescence confocal image. The imaging frame rate is determined by the camera, which is 30 frames/s in the current confocal microendoscope system. The description of the multi-spectral mode of operation of this system is described in Makhlouf *et al*. [1].

This line-scanning confocal microendoscope was modified by adding a set of relay lenses to create an image plane conjugate to the confocal slit aperture with a lateral magnification of 1.0. A custom rotating aperture is placed in the conjugate image plane shown in Figure 2. This custom multi-point aperture is a 40 mm diameter, 0.1 mm thick, tungsten disk with 360 radial slits (Δ*ϕ* = 1°) that are 23 μm wide (*w*) × 1 mm long. The radial slit width matches the slit width of the fixed confocal aperture, which produces an essentially square aperture for each intersection of a radial slit with the fixed confocal slit. The magnification between the aperture and the proximal face of the fiber bundle is 0.13, so a 23 μm × 23 μm square aperture maps to a 3 μm × 3 μm spot at the proximal fiber face, which is effectively the core size of a single fiber in the fiber bundle. The middle of each slit is located at a radial distance of 17.5 mm (*R_avg_*) from the disk center yielding a 7.5% duty cycle, or 1:13.3 ratio for aperture opening to aperture period. This choice of duty cycle was based on performance simulations that demonstrated a good tradeoff between the improvement in axial resolution and reduction in optical throughput [20].

The disk containing the radial slits is mounted to an aluminum hub that is attached to the shaft of a DC motor. The custom aperture was manufactured by Lenox Laser (Glen Arm, MD) by laser cutting through the 0.1 mm thick tungsten substrate. A mounting structure for the motor and mask was designed in SolidWorks and fabricated in FullCure 720 material on an Objet350 Connex 3D printer. The mount is such that the average radial distance of the slit apertures, *R_ave_*, is located on the optical axis and the disk axis of rotation is tilted 14° away from the optical axis. This tilt reduces the amount of reflected light that couples back into the system. The specific angle of 14° was calculated based on the numerical aperture of the line illumination beam to ensure that specular reflection from the surface of the disk falls outside the light collection angle in the detection path to the detector.

The combination of the line illumination and multi-point apertures creates a series of 18 illumination points in a line over the 0.72 mm diameter of the 30000 element fiber bundle, spaced such that approximately every 10th fiber core is illuminated. This series of illumination points is rapidly scanned in one dimension by spinning the disk. The 2D field-of-view is covered by scanning this effective line of illumination (multiple moving points along the line) in the perpendicular direction using a conventional galvanometer mirror, which is the normal line-scan approach.

In order for the multi-point architecture to work, the rotating aperture must spin sufficiently fast with respect to the number of pixels in an image and the imaging frame rate of the system. For example, our line scan confocal system operates at 30 frames/second and has an effective number of resolvable picture elements of approximately 240 × 240 based on a 3 μm fiber core size and a fiber field of view of 0.72 mm. This configuration therefore requires a slow axis sweep rate of 33ms/240 = 0.14 ms/line. The multi-point aperture must spin fast enough to cause each radial slit to travel to the location of the next radial slit (1° of rotation) within this time. This corresponds to a rotation rate of 7.2 degrees/ms, or 1200 rpm, which is readily achievable with a simple motor. The maximum allowable rotation rate is determined by the requirement that a single radial slit must not move appreciably during the time it takes light to travel from the multi-point aperture to the tissue and back. For a typical fiber bundle length of 5 meters, this round-trip time is approximately 50 ns. During this short time, the 23 μm wide radial slit on the multi-point aperture maintains 96% overlap with the return light at a DC motor rotation speed of 10,000 rpm. Thus, the frame rate could be significantly increased (by more than a factor of 8 in our system), before reaching an appreciable illumination/detection-aperture mismatch. Our imaging frame rate is currently limited by the readout rate of the camera, which operates at 30 frames per second. Practically, the amount of illumination light reaching the sample in the multi-point configuration can also be a limiting factor on the maximum frame rate due to the relatively low 7.5% transmission of the mask. Laser power can be increased to compensate for this loss, but ultimately a limit on laser power is reached and faster frame rates then lead to a decrease in the signal to noise ratio (SNR) of the acquired images.

## 3. Experimental Results

**Figure 3:**
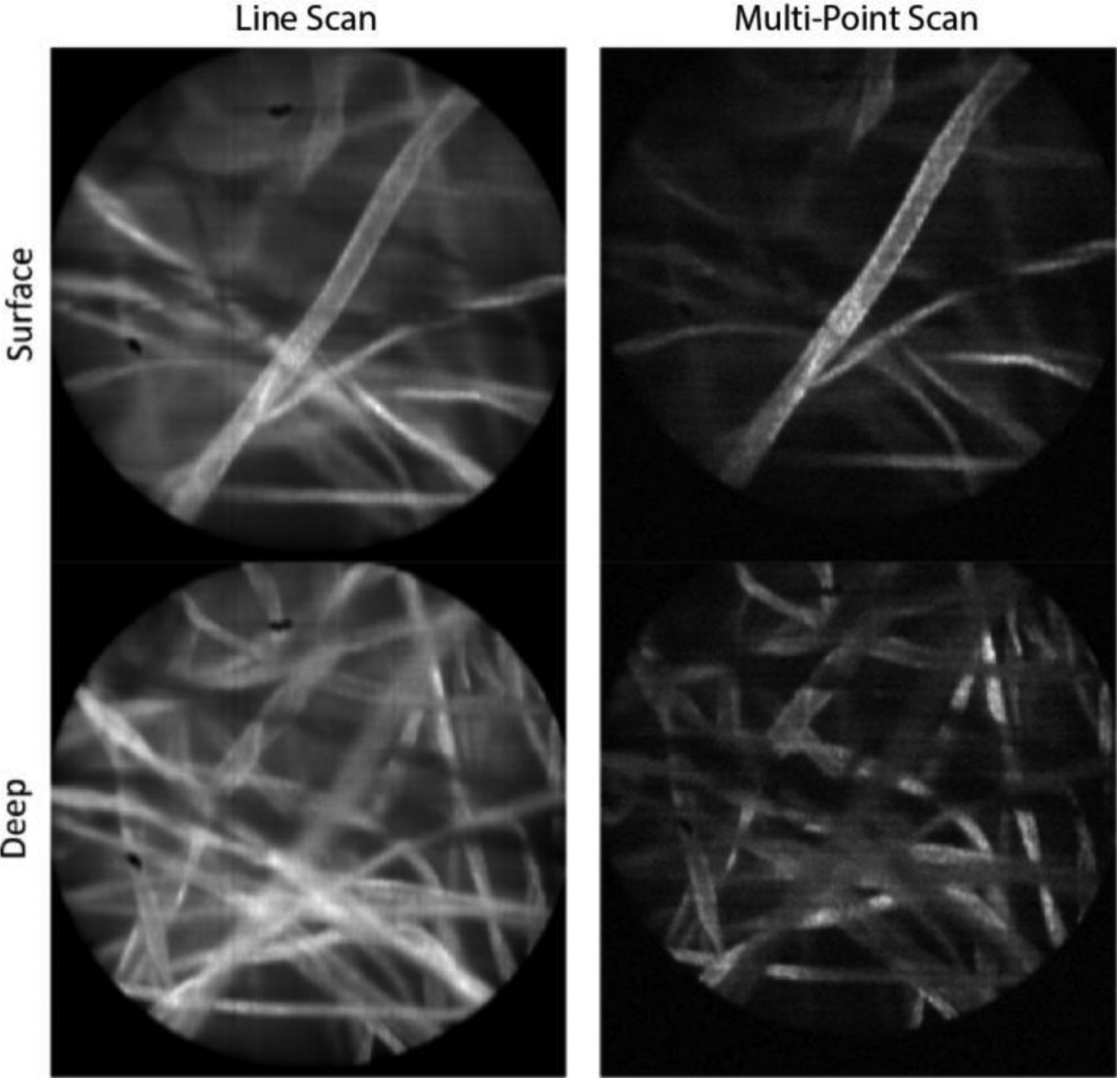
Fluorescent paper fibers imaged with both the line-scan and multi-point configurations, for object planes at the surface and at a depth of approximately 40 μm. The field of view in each image is 450 μm.

Figure 3 shows images of lens paper soaked with fluorescent dye, 330 μMolar acridine orange, taken with both the line-scan and multi-point systems. This was done for an object plane location at the surface of the paper as well as deeper within the 3D structure of paper fibers. The laser power was the same when collecting all four images, which resulted in roughly 13 times less light exposure for the multi-point images compared to the line-scan images. A lower signal value of the same factor was confirmed in the raw digital values out of the camera. For display purposes, the images in Figure 3 were normalized to the same max/min values. While the SNR in the multi-point images is decreased due to the lower exposure, the improved axial resolution of the multi-point system enables better optical sectioning, and better contrast because of the increased rejection of fluorescence signal from nearby out-of-focus paper fibers. This effect can be seen at both the surface and at the 40 μm image depth where paper fibers farther from the focal plane appear darker in the multi-point images compared to the slit-scan images.

**Figure 4:**
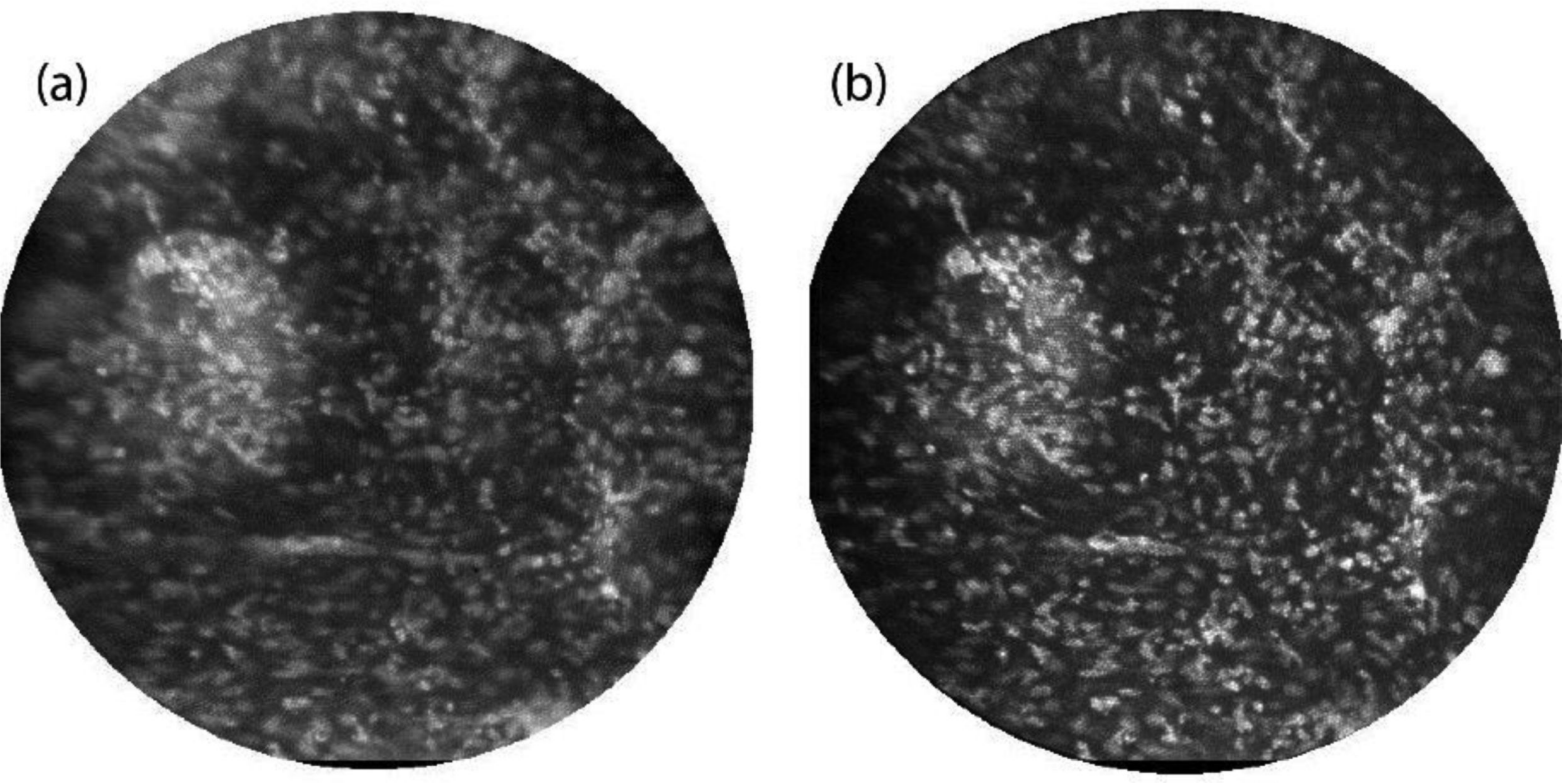
*Ex vivo* ovarian tissue stained with acridine orange and imaged by the line-scan (**a**) and multi-point (**b**) systems. The field of view in each image is 450 μm.

Figure 4 shows images of the epithelial layer of *ex vivo* human ovarian tissue topically stained with 330 μMolar acridine orange. Again, images were taken with both the line-scan (Figure 4a) and multi-point (Figure 4b) systems. In this case, the illumination laser power for multi-point scanning was increased by a factor of four over that used for slit-scan imaging. Thus the signal intensities in the bright in-focus cell nuclei are about three times lower in the multi-point image than in the line-scan image. Again the images are normalized to the same min/max values for display. Though nominally only the epithelial cell layer is stained by acridine orange, tissue surface topology can cause fluorescent signal over a range of object depths. The multi-point image has better contrast and higher axial resolution than the line-scan image, allowing better visualization of the bright punctate nuclei. A small clump of cells on the left side of the image has a lower background signal level in the multi-point image than in the line-scan image. Some improvement in lateral resolution in the bright nuclei is also noted, which is consistent with our simulations of multi-point image quality in scattering media such as tissue [20].

To determine the axial resolution performance improvement of multi-point imaging in a scattering medium, a thin fluorescent planar target was created and used to measure the axial point response of the system. The target was made by spin coating a 25 mm diameter round #1 thickness coverslip with a solution of 3 weight-% Pyrromethene 546 (Exciton, Inc.) in an equal mixture of chloroform and toluene at 1500 rpm for 15 s. The thickness of the resulting fluorescent coating was approximately 300 nm measured using a Dektak 150 surface profilometer. This coating is sufficiently thin to act as an axial planar source for testing axial resolution performance of a fluorescence confocal microscope.

To measure the axial response of the confocal microendoscope, the imaging depth was fixed at 100 μm past the end of the miniature objective lens, and the fluorescent target was submersed in mixtures of distilled water and 20% intralipid. The concentration of intralipid in each mixture was selected such that the product of the reduced scattering coefficient, $\mu_{s}^{\prime }$, and the imaging depth of the system (*d* = 100*μm*) would range from 0.1 to 2.0 (0.1 to 2.0 reduced mean free paths). The fluorescent target was then translated through focus in both line-scan and multi-point imaging modes. Images were captured for each axial translation and spatially averaged over a region of interest. These data were used to determine the axial response as a function of reduced scattering coefficient.

**Figure 5:**
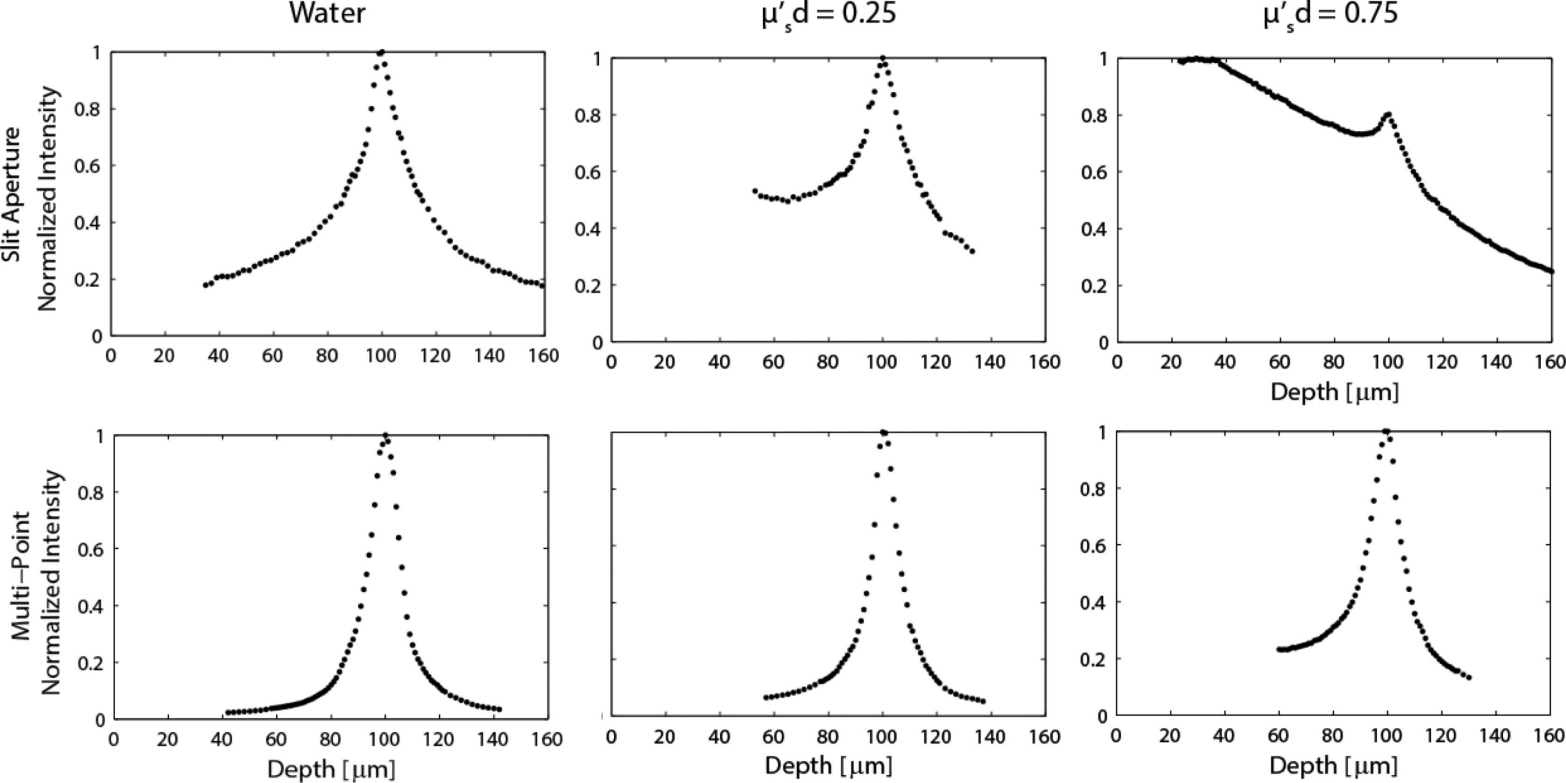
Axial response plots generated from data acquired with a thin fluorescent target for both the line-scan (top) and multi-point scan (bottom) configurations. Scattering is increased in moving from left to right. A product of $\mu_{s}^{\prime }d=1$ would indicate imaging at a depth of one reduced mean free path.

Figure 5 shows the normalized system axial response behavior for both the line-scan and multi-point systems for three solutions with different amounts of scattering. In pure water (no scattering), the slit and multi-point systems perform as expected [13,16], with measured axial intensity full-width-half-max (FWHM) of approximately 27 μm and 13.5 μm, respectively. When scattering is increased such that $\mu_{s}^{\prime }d=0.25$, the line-scan system performance is reduced to the point where it is difficult to quantify a FWHM due to the increased detection of light scattered from the media at depths between the end of the probe and the focal plane. This asymmetric response in depth is predicted in Monte Carlo simulations [22]. If the deeper half of the line-scan distribution for the $\mu_{s}^{\prime }d=0.25$ case is measured as the HWHM, the FWHM is ≈ 33 μm. If the FWHM is instead considered for both sides of the measured data as the sample is translated through focus, the FWHM ≈ 49.5 μm. By contrast, the multi-point configuration maintains the same 13.5 μm axial resolution that was calculated for the non-scattering case. When scattering is increased, such that $\mu_{s}^{\prime }d=0.75$, the line-scan signal is almost totally lost, while the multi-point depth response has only broadened slightly to a FWHM ≈ 16 μm.

## 3. Discussion

The experimental results in Figures 3 through 5 demonstrate the improved performance of the multi-point scan approach compared to the line-scan approach. The multi-point approach achieves improved axial resolution, which leads to better image contrast and an improved optical sectioning performance, especially in scattering media as predicted by Monte Carlo simulations. Theoretically the lateral resolution of the two systems should be the same for imaging in non-scattering media. However when imaging in scattering media, the lateral resolution is improved using the multi-point configuration [20]. While, this improvement in lateral resolution has not yet been quantitatively explored, we do note a qualitative improvement in lateral resolution between the multi-point and line-scan data shown in Figure 4.

Despite the performance improvements of multi-point scanning, the current implementation does have some issues. First, it was difficult to manufacture the multi-point aperture. Most laser cutting systems are programmed with a Cartesian coordinate system, which makes it difficult to accurately manufacture radial patterns. Additionally, material tends to melt and fuse to the disk during the cutting process, which leads to a non-uniform slit profile, and surface features that reflect light back into the detection path. Although this back-reflected light is filtered out by a band-reject filter at the laser wavelength, the filter is not perfect and some measurable background from the primary illumination beam does pass through the system and degrade the image quality. However, this is not a fundamental issue and image quality can be restored through more advanced mask manufacturing processes.

Second, the implemented multi-point imaging system suffers from overall poor light-throughput efficiency. Though there is no significant loss in the detected fluorescence signal for the multi-point system in comparison to the line-scan system, the duty cycle of the multi-point aperture discards 92.5% of the excitation light compared to the line-scan system. Low light levels can be overcome by increasing the laser power. However, higher illumination powers lead to larger background signals that may need to be reduced through the use of better excitation light filters in the detection path and specialized lens coatings to reduce Fresnel reflections. In addition, approaches to significantly improve illumination throughput are under development.

A key advantage of this multi-point scanning approach is its ability to maintain the capability for multi-spectral imaging inherent to a slit-scan system. The demonstration of multi-spectral imaging with the new multi-point scanning architecture is an area of continuing research. Increasing the optical throughput of the multi-point illumination system may be a necessary requirement to allow adequate frame rates with multi-spectral data acquisition.

## 4. Conclusions

We have demonstrated a novel multi-point scanning approach to confocal microendoscopy that retains the high frame rates and multi-spectral imaging capabilities of a line-scan system while improving axial resolution and image contrast. Additionally, we demonstrate that in scattering media, the multi-point imaging system performs better by rejecting more of the scattered light component, which is consistent with predictions made using Monte Carlo simulations. If manufacturing issues are addressed and a higher power source is used, future multi-point imaging systems will be capable of maintaining high imaging rates with significantly improved performance in comparison to line-scanning systems.
